# Treatment of Anterior Poliomyelitis

**Published:** 1911-04

**Authors:** Theo Toepel

**Affiliations:** Atlanta, Ga.


### TREATMENT OF ANTERIOR POLIOMYELITIS.

THEO TOEPEL, M. D., ATLANTA, GA.

It may not be amiss as an introductory remark to call your attention to the fact that this disease is spreading southward. This disease, which has prevailed since 1004 in New England and since 1907 in New York State has reached Washington, D. C. in the summer of 1910. The Medical Association of the District of Columbia convened in special session on August 23 for the consideration of the prevalence of acute anterior poliomyelitis. From the results of this investigation, which are well known to you gentlemen, I select this one sentence, referring to the treatment. "Each case may present difficulties, but these can only be met by the physician as they occur."

The most important indication in the first four to six weeks of the disease is rest in bed in order to avoid irritating the site of the lesion and that the antitoxic forces of the body may have an opportunity to limit or repel the infection. As we do not yet understand the nature of the virus or how to combat it, the treatment should be hygienic and symptomatic, the bowels should be regulated and the child made comfortable, kept well nourished and in the fresh air.

It is well known that we can favorably influence the nourishment of an affected muscle and the circulation of the blood in it by the application of electric currents, by massage and active exercise, and baths of the most varied kinds. It is well to remember when giving massage to the paralyzed muscle that it is soft and tender and that this delicate structure must be handled carefully and that the object is to strengthen it gradually.

Furthermore, as deformities, such as foot-drop, hip and knee flexion, and the like, may be acquired within a few weeks, these must be prevented by attention to posture and when necessary by moderate stretchings and splinting, but we must be careful not to overdo a good thing. Normal muscle may be injured and weakened, as we see every day by the pressure of any orthopedic apparatus, and consequently fall into a partial atrophy.

If you begin early with the orthopedic treatment and carry it out consistently, you will help the patients very much, as you will, as a rule, be able to prevent contractions.

There are patients who are so well satisfied with the result that they do not want anything further and prefer to continue it during their lives rather than to submit to an operation. Most sufferers, however, sooner or later wish to be relieved, if possible, from the apparatus which makes them dependent upon the doctor and the instrument maker. For this purpose there are several methods: redressment with or without tenotomy, transplantation of the nerves, arthrodesis and transplantation of the tendons.

Redressment is always necessary as a preliminary operation when the patient first comes under treatment when a contraction has taken place. Lorenz of Vienna, thinks that in most cases a redressment is sufficient and that in a paralytic club-foot nothing further is necessary than tenotomy redressment and a plaster of Paris cast for two months.

To repair the loss of a paralyzed muscle the nerve plastic operation has lately been frequently tried. That nerve plastic can achieve good results in many is proven by observation in cases of facial paralysis, but a result entirely free from objection in the case of a poliomyelitic patient, I have myself not yet seen. Nerve plastic in a degenerated muscle is always without result, as its warmest advocate, Dr. Spitzy acknowledges.

It is unpleasant to be obliged to stiffen a joint, to sacrifice a function of the body. Circumstances, however, often compel us to do it and when by such an operation we attain a greater ability to walk, we may not ask whether we like the operation or not. In case of the complete paralysis of the muscle of the lower leg and foot orthodesis can be a great blessing. It is well, however, not to operate before the fourteenth year, as too early an operation reduces the prospect of bony union of the joint surfaces and favors a relapse of the deformity.

If all the muscles are not paralyzed, but some strong ones are well conserved, transplantation of the tendons takes the place of orthrodesis. To-day the periosteal method is probably preferred by most orthopedists. The census taken by Dr. Lovett of Boston, was decisive in determining the opinions.

